# A review of protective effects of *Withania somnifera* and its bioactive component withaferin A in organ ischemia-reperfusion injury

**DOI:** 10.3389/fphar.2026.1961846

**Published:** 2026-09-29

**Authors:** Jiabing Wang, Nan Jin, Shihao Li, Binhui Wang, Xi Chen

**Affiliations:** 1 Department of Pharmacy, Taizhou Municipal Hospital (Taizhou University Affiliated Municipal Hospital), School of Medicine, Taizhou University, Taizhou, Zhejiang, China; 2 School of Medicine, Taizhou University, Taizhou, Zhejiang, China; 3 Zhejiang Key Laboratory of New Drug Development for Central Nervous System Diseases, Taizhou University, Taizhou, Zhejiang, China

**Keywords:** *Withania somnifera*, withaferin A, cerebral ischemia-reperfusion injury, myocardial ischemia-reperfusion injury, retinal ischemia-reperfusion injury, molecular mechanism

## Abstract

*Withania somnifera* (WS) is an Ayurvedic botanical which has been extensively studied for thousands of years. Withanolides are the main pharmacologically active ingredients isolated from WS and withaferin A (WA) is the most extensively studied and therapeutically effective withanolide compound. WS and WA have anti-ischemia-reperfusion injury effects. To summarize and investigate the protective roles of WS and WA and their underlying mechanisms in organ ischemia-reperfusion injury, a comprehensive literature search was conducted using databases including PubMed and Web of Science. Relevant *in vitro* and preclinical regarding WS and WA in the treatment of organ ischemia-reperfusion injury. By analyzing the existing research advances, we found that the anti-organ ischemia-reperfusion injury effects of WS and WA, primarily mediated through anti-apoptosis, anti-oxidative stress, anti-inflammatory, anti-mitochondrial dysfunctions, induction of neuronal regeneration and inhibition of migration. This review systematically summarizes the different roles and mechanisms of WS and WA in organ ischemia-reperfusion injury and provides a basis for future therapeutic investigation of organ ischemia/reperfusion injury.

## 1 Introduction

Ischemia/reperfusion (I/R) injury refers to a condition in which tissues or organs undergo ischemia followed by restoration of blood flow and oxygen supply (Zhang M. et al., 2024). Rather than improving organ function, reperfusion aggravates cellular metabolic disorders and damages the structure and function of cell membranes (Wu et al., 2018). I/R injury primarily affects organs with high oxygen demand, such as the heart, liver, kidneys, brain, and intestines, and is commonly observed in clinical scenarios including arterial bypass surgery, coronary angioplasty, thrombolytic therapy after vascular recanalization, cardiopulmonary bypass during cardiac surgery, and organ transplantation (Akbari et al., 2018). The pathophysiology of I/R injury is complex, often triggering local or systemic inflammatory responses and, in severe cases, leading to multiple organ failure, further complicating the condition (Kura and Slezak, 2024). Therefore, prevention and treatment of I/R injury are critical. Although many strategies have been proposed to improve perfusion, clinical outcomes remain suboptimal. Currently, ischemic preconditioning, post-ischemic conditioning, and pharmacological treatment are key approaches to mitigating I/R injury, with pharmacological interventions offering advantages in convenience and operability (Zhang M. et al., 2024). Developing effective drugs to prevent and treat I/R injury remains an important research focus.

Traditional Chinese medicines (TCMs) hold a prominent place in clinical therapy and have attracted considerable attention due to their precise efficacy, minimal adverse effects, and multi-target, multi-pathway modes of action (Li et al., 2025). In addition, TCMs are increasingly recognized as promising sources for drug discovery because of their broad spectrum of biological activities, accessibility, affordability, and structural diversity (Atanasov et al., 2021). Over 60% herbal-based products have received Food and Drug Administration (FDA) approval for market release from 1981 to 2019, while more than 100 related compounds are currently undergoing clinical trials (Newman and Cragg, 2020; Butler et al., 2026). Globally, it is estimated that 80% of the population relies on plant-based medications to fulfill therapeutic needs (Sen and Samanta, 2015). Numerous TCMs with notable pharmacological effects, along with their active constituents, have demonstrated potential in mitigating I/R injury (Wang et al., 2025; Ma et al., 2021; Zhang et al., 2025). Among these, *Withania somnifera* (WS), commonly known as Indian ginseng, is a highly valued herb with a documented history spanning nearly 3,000 years and belonging to the nightshade family (Solanaceae) (Mikulska et al., 2023). Classical Ayurvedic texts describe WS as a rejuvenating agent capable of enhancing physical and mental health, restoring vitality under pathological conditions, and promoting longevity (Wiciński et al., 2024). Over the past decade, WS extracts have been introduced into Western markets, either as isolated active compounds or as components of multi-ingredient dietary supplements, and are currently formulated in various dosage forms as herbal products (Brendler et al., 2025). WS exhibits diverse biological activities, including neuroprotective, anti-inflammatory, cardioprotective, anti-hepatitis, and antitumor effects, and has demonstrated significant efficacy in preventing ischemia-reperfusion injury in both the heart and brain (Mikulska et al., 2023; Wiciński et al., 2024; Brendler et al., 2025).

The therapeutic properties of WS are primarily attributed to a series of withanolides—polyoxygenated steroidal lactones constructed on a C_28_ ergostane skeleton—among which withanolides A and D, withaferin A (WA), and withanone are considered the most pharmacologically relevant (Williamson and Brendler, 2025; Atteeq, 2022; Paul et al., 2021; Shinde et al., 2023; Zhang Q. et al., 2024). For organ I/R injury, WA is the most extensively studied and therapeutically effective compound. This review provides an overview of the phytochemical composition and pharmacokinetic profile of WS and WA, summarizes their protective effects and underlying mechanisms in organ I/R injury (preclinical studies), including cerebral ischemia-reperfusion injury (CIRI), myocardial ischemia-reperfusion injury (MIRI), retinal ischemia-reperfusion injury (RIRI), aiming to offer a laboratory-based results for drug development, and clinical translation (Figure 1).

**FIGURE 1:**
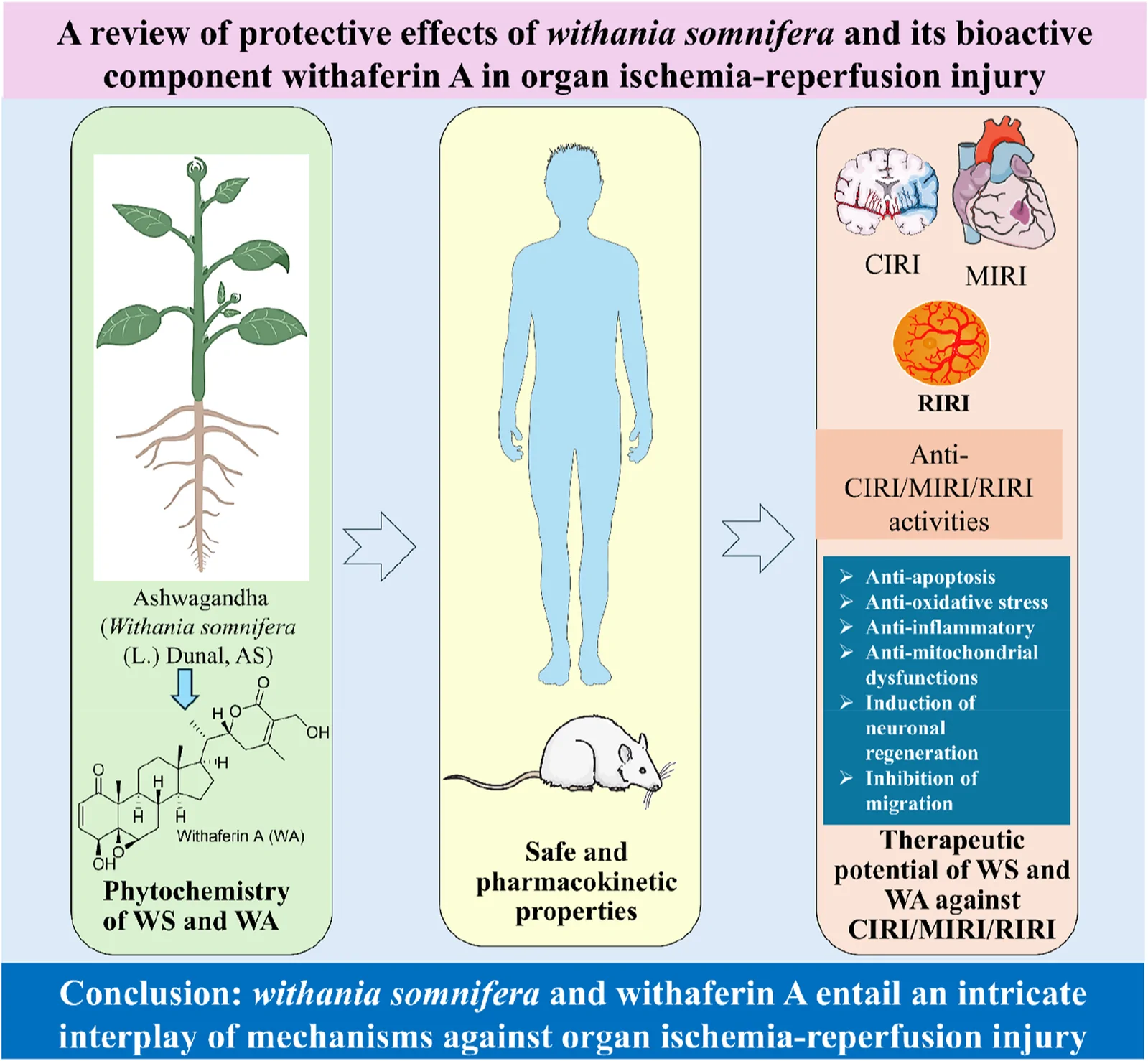
The schematic representation of this review.

## 2 The pathophysiological mechanisms of ischemia/reperfusion

I/R injury constitutes an unavoidable pathophysiological event arising from the transient interruption and subsequent restoration of organ perfusion (Zhang M. et al., 2024). Although extensive research attention has been directed toward I/R injury, the pathological mechanisms governing its initiation and progression remain incompletely elucidated. Reported pathogenic contributors to I/R injury include calcium overload, oxidative stress, mitochondrial permeability transition pore (mPTP) opening, mitochondrial dysfunction, inflammatory activation, and programmed cell death (Wang et al., 2025; Si et al., 2026; Juricic et al., 2026) (Figure 2). Consequently, the development of clinical therapeutic strategies targeting multiple molecular targets and signaling pathways in I/R injury has acquired heightened urgency.

**FIGURE 2:**
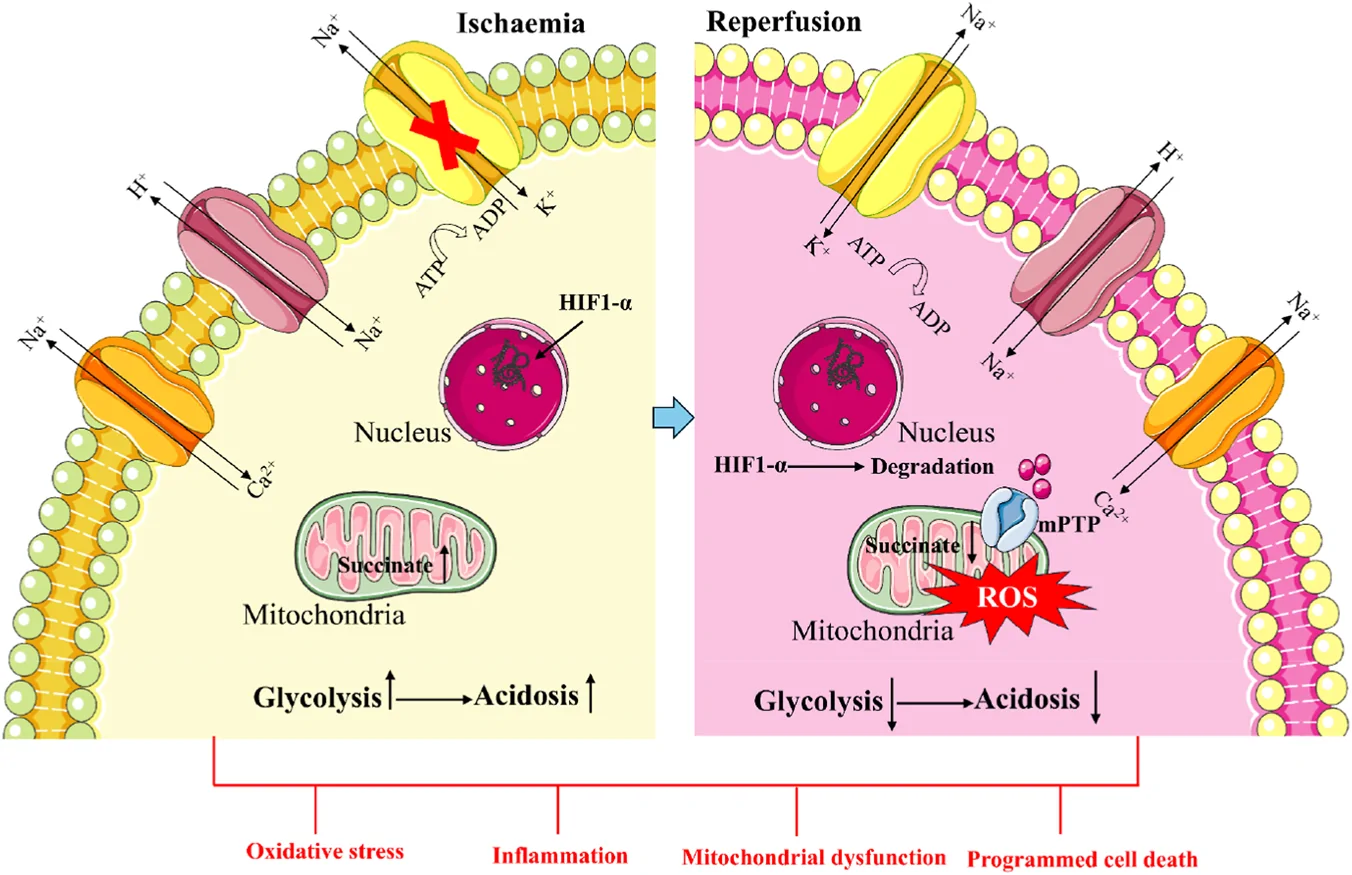
Pathology and molecular mechanisms of I/R injury. The figure illustrates the key molecular mechanisms during I/R injury. During ischemia, mitochondrial respiration is inhibited, which leads to enhanced glycolysis and the occurrence of acidosis. To balance the low pH value, the Na^+^/H^+^ exchanger (NHE) would exchange protons with Na^+^, promoting the accumulation of ions in the cytoplasm. Due to the reduced ATP supply, the activity of the Na^+^/K^+^ ATPase (NAK) is almost inhibited. Therefore, the cell uses the reverse activity of the Na^+^/Ca^2+^ exchanger (NCX) to balance the increase in Na^+^ levels. In this way, the NCX promotes the accumulation of Ca^2+^ ions in the cytoplasm while expelling sodium. At the same time, the transcription factor HIF1-α is stabilized and enters the nucleus, promoting gene expression. During reperfusion, mitochondrial respiration function is restored, thereby re-establishing the normal pH value. This rapid pH adjustment forms a large proton gradient, promoting the continuous enhancement of NHE activity, and also increasing the Na^+^ concentration in the cytoplasm. Therefore, the reverse activity of the NCX is strengthened, further increasing the Ca^2+^ level in the intracellular cavity. In this situation, Ca^2+^, together with a large amount of ROS produced by mitochondria, acts synergistically to promote the opening of the mitochondrial permeability transition pore, thereby stimulating the initiation of the cell death mechanism. In this state, HIF1-α is continuously degraded.

### 2.1 Ischemic phase

#### 2.1.1 Calcium overload

During the ischemic phase of I/R injury, affected organs undergo localized hypoxic stress, inducing substantial alterations in cellular biochemical and metabolic homeostasis (Zhang M. et al., 2024). Under hypoxic conditions, hypoxia-inducible factor 1α (HIF-1α) undergoes stabilization and nuclear translocation, thereby modulating the transcription of genes associated with angiogenesis and metabolic reprogramming (Majmundar et al., 2010). Hypoxia redirects ATP generation toward anaerobic metabolism, namely, anaerobic glycolysis (Kalog et al., 2016). Nevertheless, cellular energy requirements surpass the maximal capacity of anaerobic glycolysis, resulting in pronounced ATP depletion (Kalog et al., 2016). As an essential cofactor for numerous ATP-dependent ion transporters, diminished ATP levels severely compromise the activity of ATP-driven membrane ion pumps—including Na^+^/K^+^-ATPase and Ca^2+^-ATPase—thereby promoting lactate and hydrogen ion accumulation (Kalog et al., 2016; Perricon et al., 2014). The ensuing reduction in intracellular pH precipitates cellular acidosis. To restore intracellular pH homeostasis, the Na^+^–H^+^ exchanger (NHE) facilitates H^+^ extrusion in exchange for Na^+^ influx. Concomitant ATP depletion nearly abolishes Na^+^/K^+^-ATPase (NAK) function. As a compensatory adaptation, the Na^+^–Ca^2+^ exchanger (NCX) operates in its reverse mode to counteract elevated intracellular sodium concentrations, thereby mediating Ca^2+^ influx and Na^+^ efflux (De Lazzari et al., 2021; Chen and Vunjak-Novakovic, 2018). ATP deficiency further impairs endoplasmic reticulum Ca^2+^ sequestration and ATPase-dependent Ca^2+^ extrusion, culminating in a pathological elevation of intracellular Ca^2+^ concentration—a hallmark of calcium overload (De Lazzari et al., 2021; Bonventre and Yang, 2011).

#### 2.1.2 Mitochondrial dysfunction

In addition to ionic disequilibrium, mitochondrial dysfunction occurs concurrently during I/R injury. As the principal organelle mediating cellular aerobic respiration, mitochondria functions as the central hub for energy metabolism and represent one of the most sensitive and early-affected cellular targets during ischemic insult (Lotz et al., 2021). During the ischemic phase of I/R injury, oxygen deprivation markedly suppresses mitochondrial respiratory function and disrupts canonical metabolic pathways (De Lazzari et al., 2021). A critical metabolic alteration is the substantial elevation of the mitochondrial tricarboxylic acid cycle intermediate succinate. Such aberrant accumulation promotes intracellular acidosis and thereby exacerbates mitochondrial injury. Succinate accumulation under ischemia arises primarily from reverse operation of mitochondrial complex II, which catalyzes the conversion of fumarate to succinate upon blockade of electron flow. This process is further supported by activation of glutaminolytic pathways, which supply substrates to the tricarboxylic acid cycle and sustain succinate production (Chouchani et al., 2014; Zhang et al., 2018; Martin et al., 2019). Under hypoxic conditions, excess succinate is translocated to the cytoplasm, where it stabilizes HIF-1α and modulates the expression of downstream genes mediating adaptive responses to hypoxia (Tannahill et al., 2013; Selak et al., 2005). Concurrently, persistent deprivation of oxygen and nutrients severely impairs the mitochondrial electron transport chain (ETC). Impaired electron transfer leads to substantial electron accumulation and leakage at complexes I and III, resulting in excessive generation of reactive oxygen species (ROS) (Chen Y. et al., 2024).

### 2.2 Reperfusion phase

#### 2.2.1 Oxidative stress

During reperfusion, restoration of tissue perfusion drives rapid oxygen reintroduction, suppresses anaerobic glycolysis, relieves intracellular acidosis, and promotes progressive degradation of HIF-1α (De Lazzari et al., 2021). Although reperfusion restores essential substrates for resumption of cellular metabolism, it paradoxically amplifies abnormal ROS accumulation. Upon reoxygenation, succinate is oxidized to fumarate via mitochondrial complex II. This reaction induces reverse electron transfer (RET) through complex I from the coenzyme Q pool, enabling direct electron donation to molecular oxygen and generating large quantities of superoxide anions (Chouchani et al., 2014; Chouchani et al., 2016). Concurrently, robust Ca^2+^ influx during reperfusion induces intracellular Ca^2+^ overload, and elevated mitochondrial matrix Ca^2+^ further stimulates ROS production. This ROS burst establishes a self-amplifying positive feedback loop that initiates a cascade of pathological events and severe cellular damage, including opening of the mPTP (Morciano et al., 2017). Contemporary evidence indicates that mPTP opening does not occur during ischemia (Chouchani et al., 2014). Upon opening, mitochondrial ROS stores are abruptly released, further aggravating oxidative stress and tissue injury (Morciano et al., 2017). Elevated ROS levels are also closely associated with activation of the xanthine oxidase and NADPH oxidase systems (Granata et al., 2022).

#### 2.2.2 Inflammatory response

During the acute phase of reperfusion injury, damage-associated molecular patterns (DAMPs) released from injured cells serve as critical signals enabling the innate immune system to detect tissue damage (Chouchani et al., 2016; Lutz et al., 2010). Such DAMPs encompass intracellular molecules including high-mobility group box protein 1 (HMGB1) and heat shock proteins, which are recognized by pattern recognition receptors (PRRs) abundantly expressed on immune cell surfaces (Roh and Sohn, 2018). Among these PRRs, Toll-like receptors (TLRs)—especially TLR2 and TLR4—represent the most characterized subtypes. Engagement of DAMPs with TLRs triggers sequential intracellular signaling events that culminate in activation of the nuclear factor-κB (NF-κB) pathway. Activated NF-κB translocates to the nucleus and interacts with cognate sequences within target gene promoters, thereby driving transcriptional upregulation of diverse pro-inflammatory cytokines, including tumor necrosis factor-α (TNF-α), interleukin-1β (IL-1β), and interleukin-6 (IL-6), as well as chemokines such as monocyte chemoattractant protein-1 (MCP-1) (Alcala et al., 2022). Through chemotactic activity, these inflammatory mediators effectively promote the recruitment and directional infiltration of immune cells including neutrophils and monocytes from the peripheral circulation into injured regions, establishing prominent inflammatory cell infiltration and thereby amplifying local inflammatory responses (Slegtenhorst et al., 2014).

Concurrently, multiple metabolites generated during reperfusion, including lactate and succinate, are released into the systemic circulation. Accumulating evidence indicates that these metabolites are not merely byproducts of tissue injury, but also function as signaling molecules that act synergistically with DAMPs to modulate innate immune activation (Prag et al., 2021; Zhang et al., 2020). For instance, succinate potentiates inflammatory responses via HIF-1α stabilization or activation of G protein-coupled receptors such as GPR91 (Li et al., 2020; Chen H. et al., 2024). Progressive escalation of inflammatory signaling exacerbates tissue injury via a cascade of inflammatory-driven secondary damage, which impairs tissue functional recovery and increases the long-term risk of multiple organ dysfunction.

#### 2.2.3 Programmed cell death (PCD)

During I/R injury, oxidative stress and inflammation are not mutually independent. Instead, the two processes interact closely and reinforce one another, collectively initiating multiple forms of programmed cell death, predominantly apoptosis, pyroptosis, ferroptosis, and autophagy. Apoptosis is modulated through extrinsic death receptor pathways and intrinsic mitochondrial pathways (Elmore, 2007). Within the pathological setting of I/R injury, the intrinsic pathway serves as the core mechanism driving apoptotic initiation. Excessive ROS produced during reperfusion disrupt mitochondrial structure and function, triggering mitochondrial membrane potential depolarization and persistent abnormal opening of the mPTP. mPTP opening facilitates the release of intermembrane cytochrome C into the cytoplasm, which assembles with dATP and Apaf-1 to form apoptosomes. This complex activates caspase-9 and initiates the downstream caspase-3 signaling cascade, eventually causing DNA fragmentation and apoptotic cell death (Yang et al., 2019). Pyroptosis is tightly associated with Gasdermin D (GSDMD) (Li and Guo, 2025). In the context of I/R injury, massive DAMPs released from necrotic cells act as danger signals to activate the NLRP3 inflammasome. Activated NLRP3 recruits apoptosis-associated speck-like protein (ASC) and pro-caspase-1, facilitating autocleavage of pro-caspase-1 into enzymatically active caspase-1. Mature caspase-1 cleaves GSDMD to generate pore-forming GSDMD-N fragments that execute pyroptosis, while simultaneously promoting the maturation and secretion of proinflammatory mediators including IL-1β and IL-18 to further amplify inflammatory cascades (Yu et al., 2021). Ferroptosis represents a regulated cell death mode defined by iron-dependent ROS overaccumulation and lipid peroxidation damage (Lavie et al., 1965). Reperfusion-derived ROS initiate lipid peroxidation during I/R injury, accompanied by disrupted intracellular iron metabolism and aberrant free iron overload, which further accelerates lipid peroxide generation (Stockwell, 2022). Glutathione peroxidase 4 (GPX4) functions as a pivotal enzyme responsible for lipid peroxide elimination; its activity is markedly suppressed under I/R conditions, impairing lipid peroxide clearance and ultimately driving ferroptotic progression (Song et al., 2026). Autophagy denotes a lysosome-dependent intracellular degradation process that eliminates damaged organelles, misfolded proteins, and invasive pathogens to sustain cellular homeostasis and adapt to environmental stress (Liu et al., 2023). Moderate and transient autophagy activation at the early stage of I/R injury confers cytoprotection, for instance, through selective clearance of dysfunctional mitochondria to mitigate oxidative injury. Nevertheless, sustained oxidative stress and severe metabolic disturbance impair autophagic flux, induce abnormal accumulation of autophagic substrates, and switch autophagy from a protective adaptive mechanism to a pro-death phenotype (Decuypere et al., 2020). Overall, apoptosis, pyroptosis, ferroptosis, and autophagy do not proceed independently. These pathways collectively constitute a multilayered, dynamically regulated cell death network during I/R injury.

## 3 WS and WA: the rising active stars

### 3.1 Phytochemistry of WS and WA

Phytochemical analysis reveals that different parts of WS harbor structurally diverse and complex chemical constituents (Tewari et al., 2022). Bioactive compounds enriched in its roots, leaves, fruits, and other tissues constitute the primary material basis for its well-documented pharmacological efficacy (Tewari et al., 2022). Marked quantitative variations exist in isolated constituents between leaf and root tissues, demonstrating uneven accumulation of active ingredients across plant organs (Dipankar et al., 2025). The total chemical composition of WS extracts varies substantially with extraction solvents, including water, organic solvents, or mixed solvent systems. To date, over 12 alkaloids, 40 withanolides, and multiple sitoindosides have been isolated and experimentally validated from WS (Kumar et al., 2026). Figure 3 illustrates the characteristic constituents detected in WS roots and leaves, whereas numerous compounds in fruits remain to be further explored. Despite abundant phytochemical accumulation in roots and leaves, WS leaves and stems contain considerable cytotoxic and hepatotoxic components (Winther, 2026). Accordingly, root extracts are predominantly adopted as the major medicinal raw material.

**FIGURE 3:**
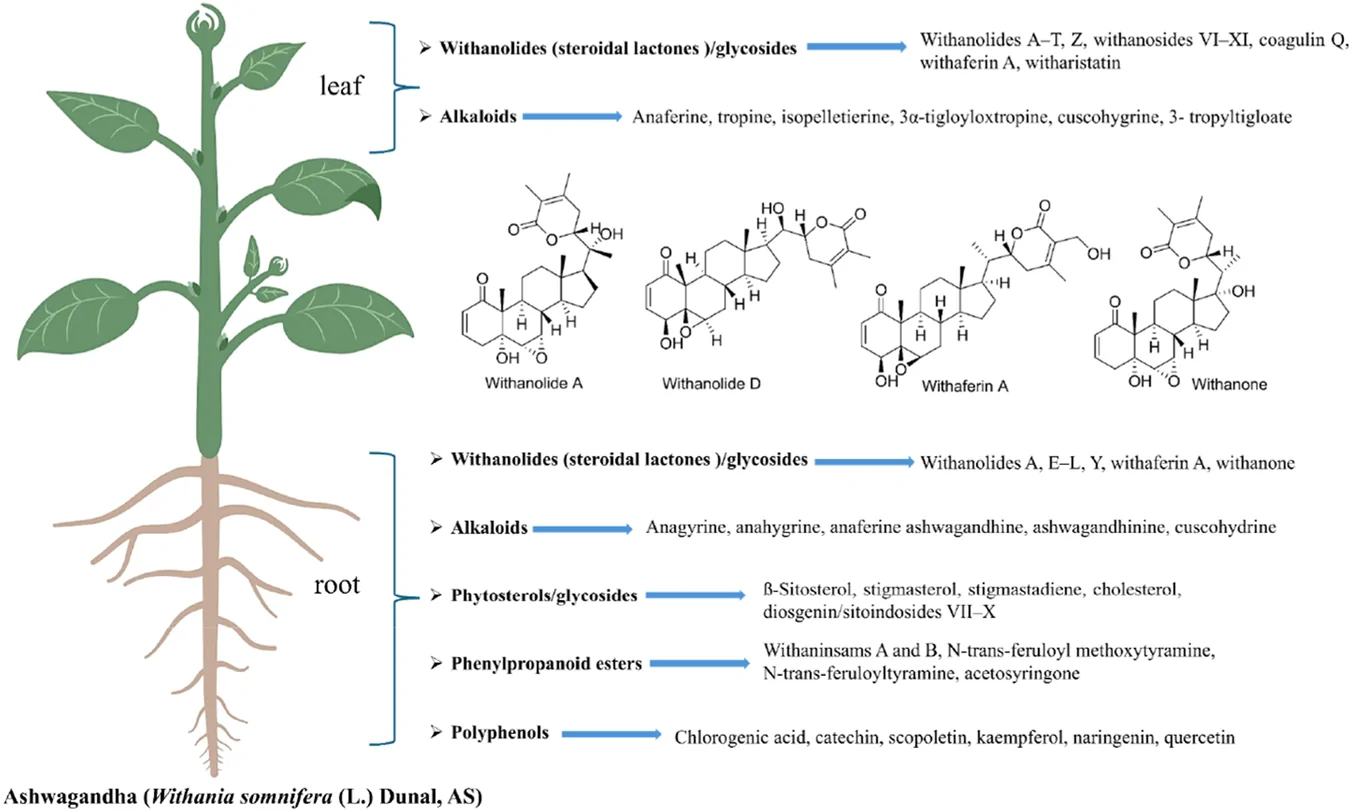
Constituents identified in *Withania somnifera* leaf and root.

Steroidal lactones (withanolides), a subclass of steroid esters, represent the principal bioactive constituents of WS, exemplified by WA, Withanolide A, Withanolide D, and Withanoside IV (Williamson and Brendler, 2025; Atteeq, 2022; Paul et al., 2021; Shinde et al., 2023; Zhang Q. et al., 2024). These compounds are regarded as the primary contributors to its pharmacological actions. Structurally, withanolides possess a C28 ergostane-type steroid skeleton, featuring a lactone ring at the C22 position and a hydroxyl group at the C26 position; both structural moieties are critical to biological activity (Williamson and Brendler, 2025; Atteeq, 2022; Paul et al., 2021; Shinde et al., 2023; Zhang Q. et al., 2024). Among all withanolides, WA was first isolated and identified from WS in 1965 (Lavie et al., 1965). Structurally, WA (C_28_H_38_O_6_) consists of four cycloalkane rings, and among which three are cyclohexane and one is cyclopentane (Atteeq, 2022; Kumar et al., 2024). There are two hydroxyl groups, one in the A ring of the ergostane framework and the other in the delta lactone side chain (Atteeq, 2022; Kumar et al., 2024). The α,β-unsaturation in the A ring, 5β,6β-epoxy in the B ring and lactone side chain in the E ring are considered to be prone to nucleophilic attack, and all these sites form the basis for the strong biological activity of WA (Figure 4A) (Atteeq, 2022; Kumar et al., 2024; Kumar et al., 2023). In addition, WA possesses the drug-likeness properties because it complies with Lipinski’s rule (Nendza et al., 2010). A summary of the physicochemical properties of WA is presented in Figure 4B. The data in the table are available at https://www.swissadme.ch/ and https://pubchem.ncbi.nlm.nih.gov. Furthermore, it has since been reported to exert multiple biological properties, including neuroprotection, anticancer, anti-inflammatory, anti-diabetic and cardioprotective activities (Sultana et al., 2021; Abeesh and Guruvayoorappan, 2024; Bungau et al., 2021). Furthermore, WA stands as the most extensively investigated monomer compound, exhibiting prominent therapeutic potential against organ I/R injury.

**FIGURE 4:**
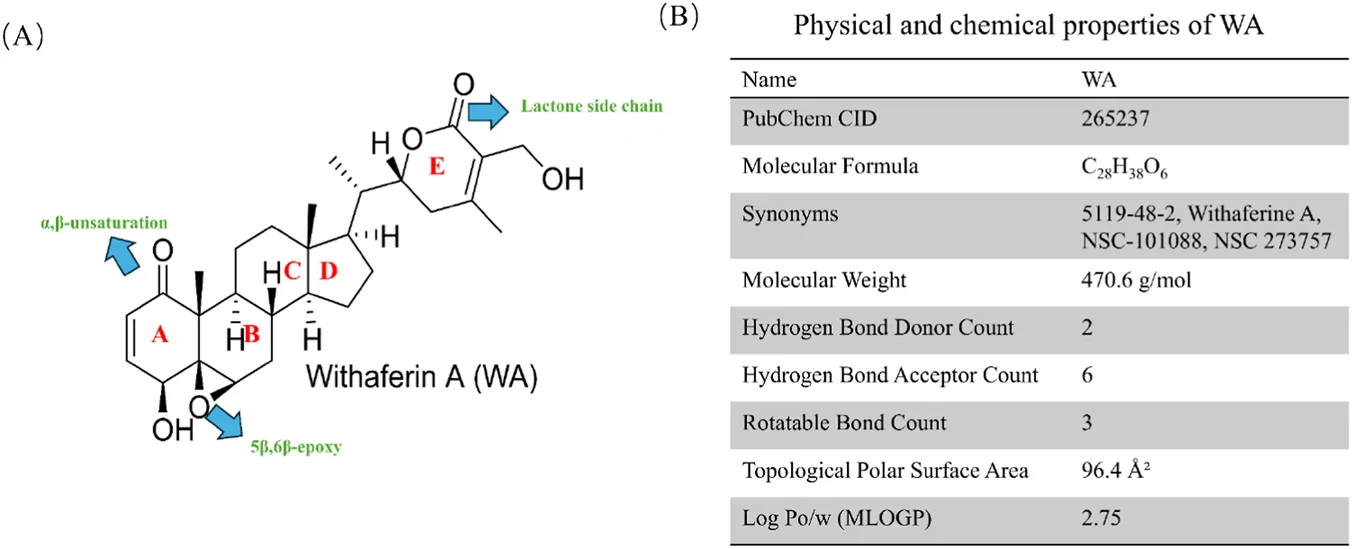
The structure **(A)** of and physical and chemical properties **(B)** of WA.

### 3.2 Safety studies of WS and WA

Accumulating experimental evidence has validated the safety of WS extract and WA, with no overt toxic effects identified. In acute toxicity assays, oral administration of WS root extract (WSC) and WA at doses up to 2,000 mg/kg was well tolerated in rodents, with no toxic symptoms or mortality observed (Antony et al., 2018; Gurav et al., 2023; Patel et al., 2016; Prabu and Panchapakesan, 2015; Prabu et al., 2013). This dose corresponds to an estimated 22 g daily human intake, far exceeding the routine recommended dosage of WS extract (Williamson and Brendler, 2025). In Swiss albino mice receiving intraperitoneal injection of WSC, the no observed adverse effect level (NOAEL) exceeded 1,100 mg/kg, while the median lethal dose (LD_50_) was recorded at 1,260 mg/kg (Sharada et al., 1993). Subacute toxicity evaluations in rodents further confirmed the absence of toxic responses. A 28-day subacute toxicity study in Wistar rats treated with standardized WSC (KSM-66) at 200, 400, and 800 mg/kg revealed no abnormal pathological alterations across all detected indicators (Langade et al., 2023). Gupta et al. conducted a 28-day subacute toxicity trial via oral WA administration in mice at doses of 10, 70, and 500 mg/kg (Gupta et al., 2022). No dose-dependent drug toxicity was detected at any concentration, establishing a NOAEL of at least 500 mg/kg (Gupta et al., 2022). A 30-day repeated intraperitoneal subacute toxicity study also reported no toxic manifestations. Subchronic and chronic investigations further corroborate the high safety profile of WS. Three independent 90-day subchronic studies yielded an average NOAEL of 1,333 mg/kg per day (Antony et al., 2018; Kalaivani et al., 2023; Wangikar et al., 2024). In a chronic trial, albino rats received daily oral WS root decoction at 100 mg/kg for eight consecutive months; no obvious toxic effects were noted apart from minor body weight fluctuations (Sharma et al., 1985). Although isolated adverse events including gastrointestinal discomfort, immune hypersensitivity, potential hepatotoxicity and endocrine disturbances have been documented following long-term or excessive intake of WS or WA, such sporadic cases do not negate the overall favorable safety profile of WS in general populations (Kumar et al., 2026). Collectively, available toxicological data support the safe application of WS and WA as herbal medicinal agents and justify further in-depth research.

### 3.3 Pharmacokinetic properties of WS and WA

Pharmacokinetic evaluation is essential for elucidating the action mechanism and guiding safe application of exogenous compounds (Zhou et al., 2011). Accordingly, the absorption, distribution, metabolism, and excretion (ADME) profiles of WS and WA are discussed (Figure 5).

**FIGURE 5:**
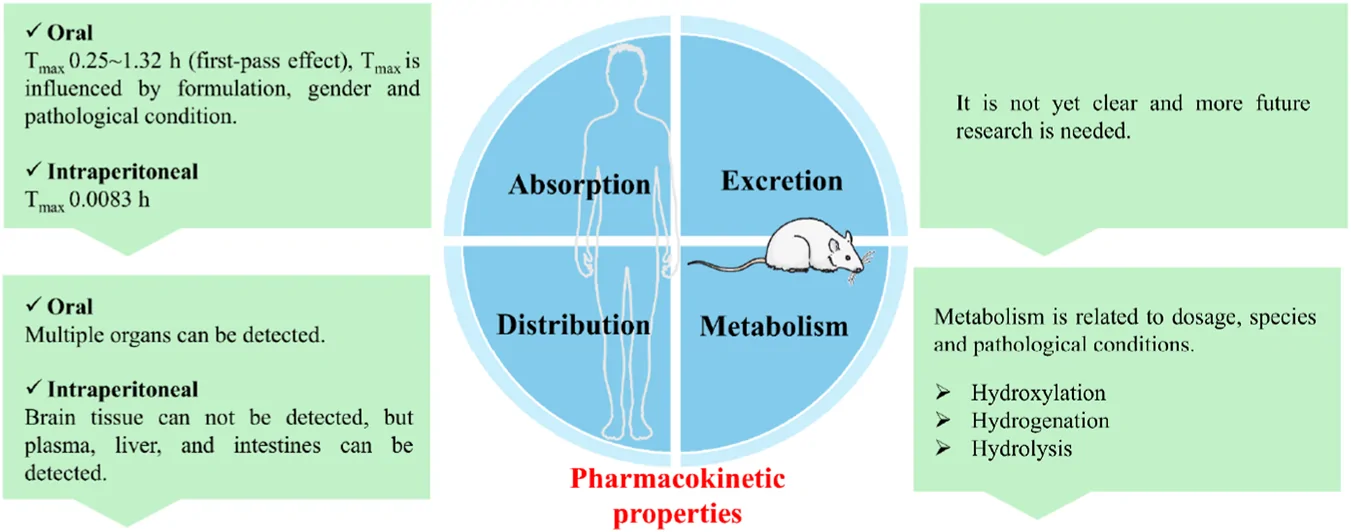
The schematic representation of WS and WA pharmacokinetic properties.

#### 3.3.1 Absorption

Two investigations in Swiss albino mice examined oral administration of hydroalcoholic (80:20) WSC at 200 mg/kg and aqueous WSC at 1 g/kg (Srivastava et al., 2013; Patil et al., 2013). For WA, the corresponding T_max_ values were 1.32 h and 20 (20–30) min, with C_max_ reaching 60 ng/mL and 16.69 ± 4.02 ng/mL, respectively (Gurav et al., 2023; Patel et al., 2016). The respective ${\text{AUC}}_{0-\infty }$ values were 11.50 ng/mL·h and 1,673.10 ± 54.53 ng/mL·min (Gurav et al., 2023; Patel et al., 2016). Three independent rat studies using withanolide-enriched plant extracts detected WA in plasma as early as 15 min post-administration, demonstrating rapid *in vivo* absorption (Modi et al., 2022; Dadge et al., 2023; Muhasaparur Ganesan et al., 2021). Plasma pharmacokinetic analysis following single WA dosing in mice and rats revealed pronounced variations in absorption-related parameters across different dosages, administration routes, and animal models. The oral absorption of WA occurs moderately rapidly, with T_max_ ranging from 0.25 to 1 h in mice (Langade et al., 2023); however, T_max_ is prolonged to 3–4 h in estrogen-deficient BALB/c mice with bone loss, implying that pathological status interferes with intestinal absorption (Khedgikar et al., 2013), suggesting that the pathological condition affects the absorption of WA. The absorption rate of intraperitoneal administration in WA is extremely fast (T_max_ = 0.083 h) in mice, closely resembling the pharmacokinetic profile of intravenous delivery (Thaiparambil et al., 2011). Relative to intravenous injection, oral dosing produces a markedly lower C_max_, indicative of limited the oral absorption efficiency of WA (Langade et al., 2023; Thaiparambil et al., 2011).

Pharmacokinetic traits of WS and WA have also been characterized in human trials. In Pires et al. (2020) performed a phase I dose-escalation study employing WA doses of 72, 108, 144, and 216 mg in patients with advanced high-grade osteosarcoma; nonetheless, WA was undetectable in peripheral blood across all subjects, potentially attributable to the low sensitivity of ultraviolet spectroscopic detection. Alluri et al. (2022) compared aqueous WSC with sustained-release capsules formulated from the same extract and confirmed enhanced withanolide bioavailability with the sustained-release preparation. In Vaidya et al. (2024) conducted a single-dose trial in 18 healthy males using 500 mg capsules of hydroalcoholic WSC standardized to 1.5% total withanolides (7.5 mg). The measured C_max_ of withanoside IV, withanolide A, WA, and 12-deoxywithastramonolide were 0.639 ± 0.211, 2.926 ± 1.317, 2.833 ± 0.981, and 5.498 ± 1.986 ng/mL, while corresponding T_max_ values were 1.639 ± 0.993, 1.361 ± 0.850, 0.903 ± 0.273, and 1.375 ± 0.510 h. These findings support rapid gastric absorption and favorable oral bioavailability of withanosides and withanolides, which appears inconsistent with earlier reports stating absolute bioavailability below 5%. A 2025 study by Sharma et al. (2025) reported that, following oral intake of 400 mg WSC capsules (2.5% total withanolides) in 12 participants, the detectable duration of withanolide A and WA in male plasma reached 15 h and 12 h, respectively, considerably longer than the 6 h observed in females, suggesting gender-dependent absorption characteristics. Recent research further evaluated oral pharmacokinetics and plasma bioequivalence of bioactive withanolides from four commercial WSC formulations (WS-35, WS-10, WS-5, and WS-2.5). Among these preparations, WS-35 displayed distinctly higher C_max_ and superior bioavailability, highlighting high concentrations of withanolide glycoside could significantly increase the total exposure level and its clinical priority potential (Rathi and Kim, 2025).

#### 3.3.2 Distribution

Modi et al. (2022) performed *in vitro* assessments on the major bioactive constituents of WS to predict their blood–brain barrier (BBB) permeability. Withanolide A, withanolide B, withanone, 12-deoxywithastramonolide, and WA exhibited BBB penetrability, whereas withanoside IV and V failed to cross the barrier (Modi et al., 2022). Such disparity is presumably attributed to their relatively high molecular weight (>700 Da) (Levin, 1980; Banks, 2009). Dadge et al. (2023) administered a withanolide-enriched hydroalcoholic root extract fraction at 50 mg/kg to male Sprague–Dawley rats and monitored compound concentrations in brain homogenates over 8 h. Notably, WA maintained peak brain concentrations for up to 2 h (Dadge et al., 2023). By contrast, Gambhir et al. delivered WA via intraperitoneal injection at 50 mg/kg to Swiss albino mice and examined tissue distribution within 4 h; WA was undetectable in brain tissue but present in plasma, liver, and intestines (Gambhir et al., 2015). In another study, Wang et al. (2019) orally administered 4.5 mg/kg WA to Sprague–Dawley rats and quantified tissue levels within 5 h, detecting WA in multiple visceral organs, including the stomach, intestines, heart, liver, lungs, kidneys, and spleen.

#### 3.3.3 Metabolism

Cytochrome P450 (CYP450) represents a family of membrane-bound proteins predominantly located on the endoplasmic reticulum of mammalian cells, with the highest abundance in the liver and small intestine (Manikandan and Nagini, 2018). These isoforms exert pivotal roles in physiological processes, particularly drug metabolism, by catalyzing redox reactions such as oxidation, reduction, hydroxylation, and dealkylation (Manikandan and Nagini, 2018). As the primary phase I metabolic enzyme system in humans, CYP450 participates in the biotransformation of most herbal ingredients (Rendic and Guengerich, 2015; Fasinu et al., 2012). Nevertheless, existing studies have reported inconsistent findings regarding the regulatory effects of WS and WA on CYP450 activity. Dai et al. proposed that WA undergoes extensive first-pass metabolism, as it serves as a substrate for P-glycoprotein and microsomal CYP3A4 (Dai et al., 2019). Conversely, Savai et al. (2014) and Savai et al. (2015) observed no notable interactions between WSC and CYP1A, CYP2D6, CYP1A2, or CYP2C9 in rat and human liver microsomes. Discrepancies across studies may arise from variations in experimental design as well as extract compositional differences. The elimination half-life (t_1/2_) of oral WA ranges from 0.86 to 2.7 h in BALB/c mice (70 mg/kg) (Gupta et al., 2022) and Sprague–Dawley rats (0.5, 1.5, 4.5 mg/kg) (Wang et al., 2019). In contrast, oral WA at 10 mg/kg yielded t_1/2_ values of 7.6 h and 7.1 h in Sprague–Dawley rats (Dai et al., 2019) and estrogen-deficient bone-loss BALB/c mice (Khedgikar et al., 2013), respectively. Despite ongoing controversies over WA metabolic patterns, available evidence indicates that WA is primarily metabolized in rats through hydroxylation, hydrogenation, and hydrolysis pathways (Dai et al., 2019).

#### 3.3.4 Excretion

Currently, no published studies have investigated urinary and fecal excretion profiles or performed metabolic profiling following WS or WA administration in either animal or human models. This represents a critical research gap that requires urgent exploration.

## 4 Pharmacological effects and mechanisms of WS and WA in organ ischemia-reperfusion injury

With the expanding clinical adoption of reperfusion strategies, organ ischemia-reperfusion injury has emerged as a critical determinant dictating prognostic outcomes in ischemic disorders. Characterized by inherent medicinal and edible homology, TCM exerts multi-target regulatory actions via abundant natural bioactive constituents, thereby supporting long-term application for the prophylactic management of organ ischemia-reperfusion injury. Accumulative investigations have elucidated the broad pharmacological properties of WS and WA, with growing attention directed toward their therapeutic potential against ischemia-reperfusion-induced organ damage. Table 1 and Figures 6–8 showed the mechanisms of WS and WA in cerebral/myocardial/ retinal ischemia-reperfusion injury.

**TABLE 1:** The research progresses of effects and mechanisms of WS and WA against CIRI, MIRI and RIRI.

Disease	WS/WA	Dosage and administration method	Model	*In vitro*/*in vivo*	Upregulation	Downregulation	Mechanism	References
CIRI	WS	100 μg/mL (*in vitro*), 200 mg/kg po (*in vivo*)	H_2_O_2_ (*in vitro*), pMCAO (*in vivo*)	*In vitro* (PC12 cells)/*in vivo* (C57BL/6 mice)	HO-1	PARP1, AIF, Sema 3A	WS improves CIRI by attenuating PARP1-AIF-mediated caspase-independent apoptosis	Raghavan and Shah (2015)
	WS	300 mg/kg po	MCAO (*in vivo*)	*In vivo* (Male wistar rats)	LMW-SHs, T-SHs, P-SHs	MDA	WS attenuates CIRI against oxidative stress, behavioural, biochemical and histological changes	Sood et al. (2016)
	WS	1,000 mg/kg po	MCAO (*in vivo*)	*In vivo* (Male wistar rats)	—	MDA	WS attenuates CIRI through ameliorating oxidative stress	Chaudhary et al. (2003)
	WS	300 mg/kg po	MCAO (*in vivo*)	*In vivo* (Male wistar rats)	SOD, CAT, GSH, GSSG, GSH/GSSG, NADH dehydrogenase, Succinate dehydrogenase, Cytochrome oxidase, F1-F0 synthase, MTT reduction, Mitochondrial membrane potential, complex-I, complex-II, complex-III + IV, complex-IV, complex-V, Bcl-2	ROS, Protein, Carbonyls, Caspase-3, Caspase-9, Bax	WS attenuates CIRI through ameliorating oxidative stress and mitochondrial dysfunctions	Sood et al. (2018)
	WS	300 mg/kg po	MCAO (*in vivo*)	*In vivo* (Male wistar rats)	GABA, NE	IL-6, TNF-α, MCP-1, COX-2, NF-κB, glutamate, DA, 5-HT	WS attenuates CIRI through anti-inflammatory response and modulation of neurotransmitter	Sood et al. (2024)
	WA	75, 150, 300 μg/mL (*in vitro*), 25, 50, 100 mg/kg ig (*in vivo*)	PDGF (*in vitro*), MCAO (*in vivo*)	*In vitro* (A7r5 cells)/*in vivo* (SD rats)	p-PI3K, p-Akt, Bcl-2, Bcl-xL, GSK-3β, cyclinD1, mTORc1,	MMP-2, MMP-9, Bax, Bad, Caspase-3, PTEN, NF-κB	WA attenuates CIRI by activating the PI3K/Akt pathway, modulating the expression of MMPs and inhibiting the migration of VSMCs	Zhang et al. (2017)
MIRI	WS	50 mg/kg po	LCAO (*in vivo*)	*In vivo* (Wistar rats)	SOD, GSH, CAT, GSHPx	MDA	WS attenuates MIRI through antioxidant	Gupta et al. (2004)
	WS	50 mg/kg po	LCAO (*in vivo*)	*In vivo* (Wistar rats)	GSH, CPK, Bcl-2	TBARS, Bax	WS attenuates MIRI through antioxidant and anti-apoptosis	Mohanty et al. (2008)
	WA	25, 50, 100, 200 nM (*in vitro*), 100, 1,000 nM (*in vivo*)	H_2_O_2_ (*in vitro*), LCAO (*in vivo*)	*In vitro* (NRVMs and H9c2 cells)/*in vivo* (wild-type mice)	SOD2, SOD3, Prdx1, Akt	Caspase-3	WA attenuates MIRI through activated Akt-mediated inhibition of oxidative stress	Yan et al. (2018)
	WA	1, 5 mg/kg ip	LCAO (*in vivo*)	*In vivo* (wild-type and AMPK-DN transgenic mice)	Bcl-2, p-AMPK, p-ACC, p-P38, p-JNK, p-ERK1/2	Caspase-3, Caspase-9, Bax,	WA attenuates MIRI through AMPK-dependent upregulation of the mitochondrial anti-apoptotic	Guo et al. (2019)
RIRI	WA	12.5, 25, 50, 100 nM (*in vitro*), 10 nmol/g ip (*in vivo*)	H_2_O_2_ (*in vitro*), PPAO/ECAO (*in vivo*)	*In vitro* (HRMECs)/ *in vivo* (Male C57Bl/6J mice)	HO-1, Prdx1, Akt	LDH, Caspase-3	Withaferin attenuates RIRI via Akt-dependent inhibition of oxidative Stress	Yan et al. (2022)

**FIGURE 6:**
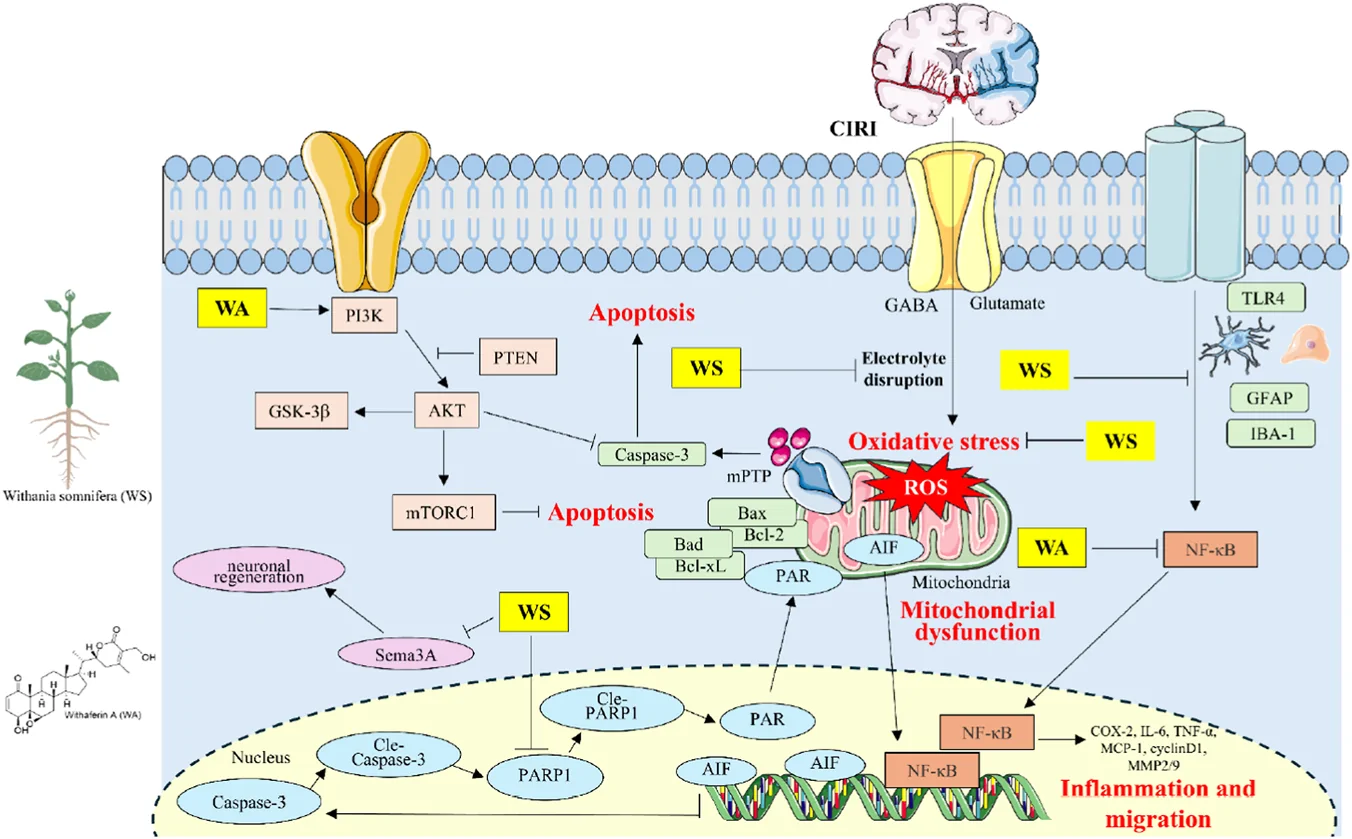
The protective mechanism of WS and WA against CIRI. WS and WA. The protective mechanisms of WS and WA against CIRI encompass the following aspects: alleviating oxidative stress, inhibiting inflammation, suppressing mitochondrial dysfunctions and apoptosis, promoting neuronal regeneration, and inhibiting vascular smooth muscle cell (VSMC) migration.

**FIGURE 7:**
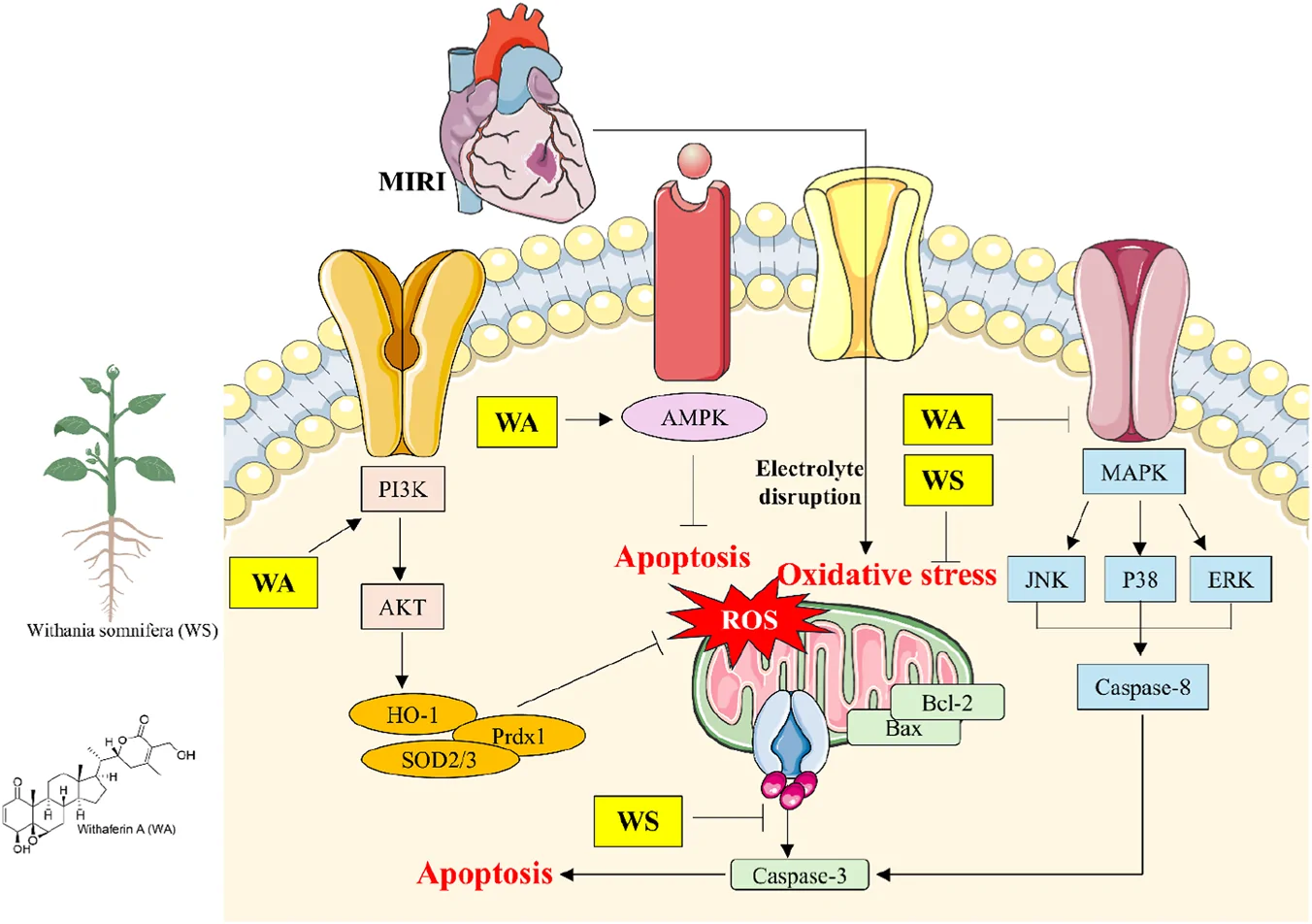
The molecular mechanisms of WS/WA against MIRI. The protective mechanisms of WS and WA against CIRI encompass the following aspects: alleviating oxidative stress and suppressing apoptosis.

**FIGURE 8:**
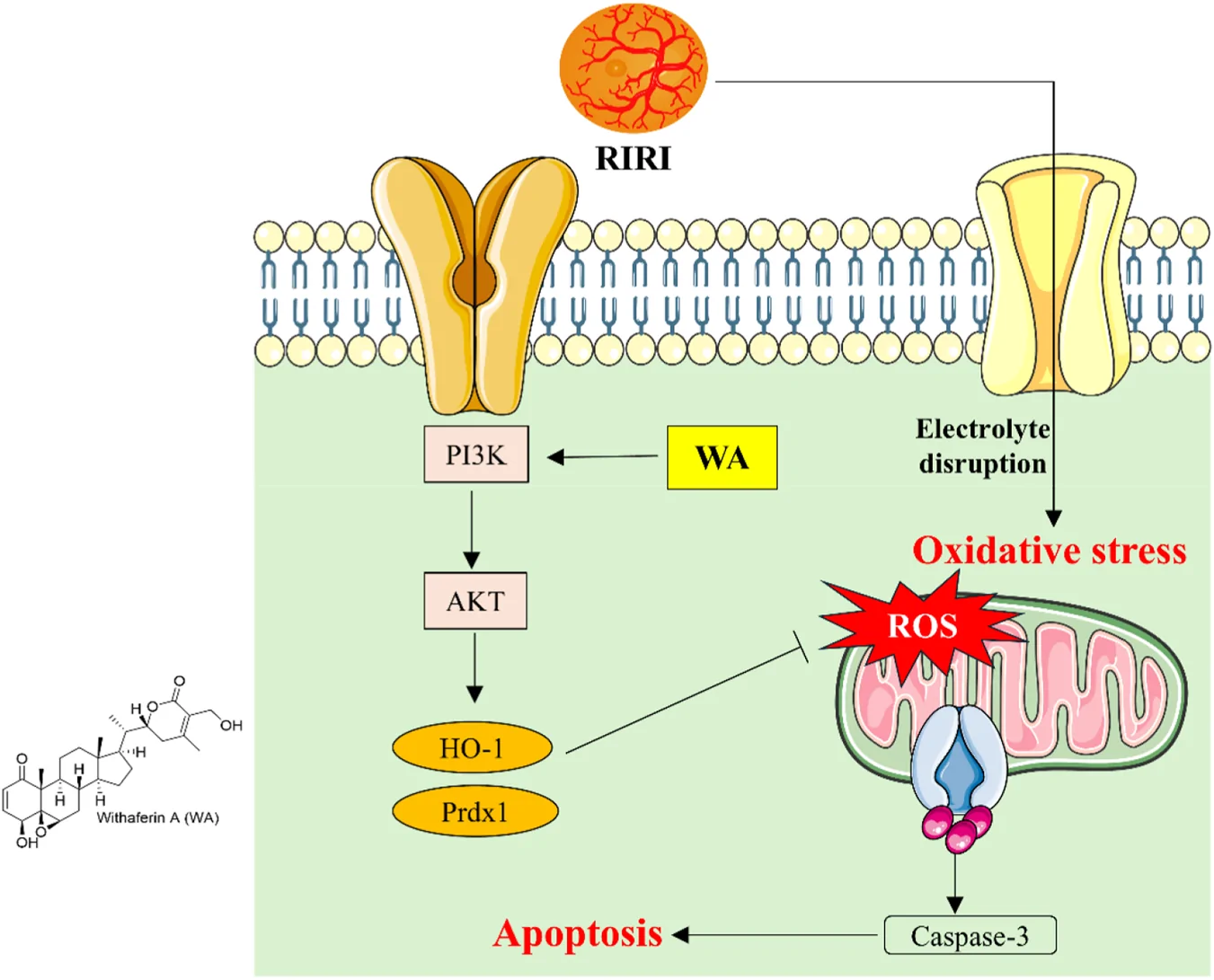
The molecular mechanisms of WA against RIRI. The protective mechanisms of WA against RIRI via the Akt-dependent Prdx-1 and HO-1 antioxidant signaling pathways and caspase-3 mediated apoptosis.

### 4.1 Effects and mechanisms of WS and WA against CIRI

The brain has high metabolic activity and requires a large amount of oxygen, and thus, it is particularly sensitive to hypoxia and subsequent reperfusion injury. Stroke interrupts the blood supply to the arteries, resulting in a sharp reduction in ATP synthesis, and immediately impairs the function of ion pumps, disrupting cellular homeostasis and triggering cytotoxic edema (Chi et al., 2026). Although the commonly used thrombolytic and thrombectomy procedures can restore blood supply, they may trigger secondary damage. During reperfusion, the restoration of blood flow can trigger a series of pathological events such as oxidative stress, inflammatory cascade, excitatory amino acid toxicity, and calcium overload, which further damage the integrity of the blood-brain barrier, accelerate neuronal apoptosis, and ultimately exacerbate neurological deficits (Chi et al., 2026). Researches have confirmed that WS and WA have potential activity against CIRI through alleviating oxidative stress, inhibiting inflammation, suppressing mitochondrial dysfunctions and apoptosis, promoting neuronal regeneration, and inhibiting vascular smooth muscle cell (VSMC) migration (Figure 6).

#### 4.1.1 Alleviating oxidative stress

The property of alleviating oxidative stress of WS has been validated in murine models with permanent distal middle cerebral artery occlusion (pMCAO) through both prophylactic and therapeutic interventions in research by Raghavan and Shah (2015). In relevant trials, mice received daily oral administration of 200 mg/kg aqueous WSC for seven consecutive days prior to pMCAO modeling, or equivalent WS treatment initiated at 4 hours post-pMCAO and sustained for another 7 days. Both pretreatment and post-treatment regimens of WS markedly ameliorated histological lesions and short-term neurological functional deficits. Mechanistic investigations further demonstrated that the protective capacity of WS is closely associated with heme oxygenase-1 (HO-1)-dependent antioxidant responses (Raghavan and Shah, 2015). The anti-oxidative stress efficacy of WS pretreatment for 30 days was further assessed in a transient ischemic stroke model established via middle cerebral artery occlusion (MCAO) in rats (Sood et al., 2016; Chaudhary et al., 2003; Sood et al., 2018). The results showed that the marker of oxidative stress malondialdehyde (MDA) was significantly decreased compared with vehicle-treated rats (Sood et al., 2016; Chaudhary et al., 2003), while the levels of total thiols (T-SHs), low molecular weight thiols (LMW-SHs) and protein thiols (P-SHs) were elevated under WS pre-supplementation (Chaudhary et al., 2003). Besides, significant decrease in oxidative stress was observed in terms of decreased levels of ROS and protein carbonyls when pre-supplementation with WS to MCAO animals, but the activities of antioxidant enzymes glutathione (GSH), superoxide dismutase (SOD), catalase (CAT) including redox (GSH/GSSG) ratio were found to be significantly increased in MCAO animals with WS pre-supplementation (Sood et al., 2018).

#### 4.1.2 Inhibiting inflammation

Neuroinflammation has been identified as one of the main factors influencing the prognosis of CIRI, and inhibiting the inflammatory response in CIRI has a positive effect (Talma et al., 2016). Sood et al. (2024) found that the level of IL-6, TNF-α, MCP-1, cyclooxygenase-2 (COX-2) were increased in the MCAO animals. However, supplementation of WS to MCAO animals significantly decreased the activity of inflammatory mediators such as IL-6, TNF-α, MCP-1 and COX-2, suggesting reduction in neuroinflammation in WS presupplemented MCAO animals, and one mechanism by which WS may function is through the NF-κB pathway (Sood et al., 2024).

#### 4.1.3 Suppressing mitochondrial dysfunctions and apoptosis

Mitochondria are the primary source of ROS within cells and are regarded as one of the main targets of CIRI. Regulating mitochondrial dysfunction is an effective way to treat CIRI. Researches have found that the mitochondrial respiratory chain enzymes such as NADH dehydrogenase, succinate dehydrogenase, cytochrome c oxidase and F1–F0 synthase, mitochondrial complex-I, complex-II, complex-III + IV, complex-IV and complex-V were found to be significantly increased under WS treatment (Sood et al., 2018). Meanwhile, mitochondrial swelling and mitochondrial membrane potential were found to be significantly decreased and increased in MCAO animals pre-supplemented with WS respectively, accompanied by the restoration of mitochondrial morphology (Sood et al., 2018). Apoptosis is a major event following the impairment of mitochondrial respiratory chain function in I/R injury. Interestingly, WS and WA pre-supplementations to MCAO animals significantly reduced the activity of the cytosolic caspase 3 and caspase 9, and reversed the expression of Bcl-2 and Bax proteins as compared to MCAO group (Sood et al., 2018; Sood et al., 2024). Moreover, poly (ADP-ribose) polymerase-1 (PARP1)/apoptosis-inducing factor (AIF) and phosphoinositide 3-kinase (PI3K)/protein kinase B (Akt) signaling pathway are involved in the anti-apoptotic effect of WS and WA, respectively (Raghavan and Shah, 2015; Zhang et al., 2017).

#### 4.1.4 Promoting neuronal regeneration

Neuronal repair plays a crucial role in enhancing the clinical treatment outcome and accelerating the overall recovery process of patients with CIRI. Semaphorin-3A (Sema3A) is an important member of the semaphorins family, which is known to inhibit axon growth and induce growth cone collapse (Kolodkin et al., 1993; Luo et al., 1993). Moreover, the level of Sema3A increases following stroke, suggesting Sema3A may be involved in the inhibitory mechanism leading to brain damage (Raghavan and Shah, 2015). Raghavan et al. found that compared with the control group mice, the expression level of Sema3A in the cerebral cortex of mice treated with WS was decreased, and the reduced levels of Sema3A are beneficial for neuronal repair (Raghavan and Shah, 2015).

#### 4.1.5 Inhibiting VSMC migration

*In vitro* experiments using platelet-derived growth factor (PDGF)-stimulated A7r5 cells indicated prominent suppression of matrix metalloproteinase (MMP)-2 and MMP-9 activities following WA exposure, suggesting WA treatment restrained A7r5 cell migration (Zhang et al., 2017).

### 4.2 Effects and mechanisms of WS and WA against myocardial ischemia-reperfusion injury (MIRI)

Current understanding suggests that the pathophysiology of MIRI is intimately linked to oxidative stress, calcium overload, mitochondrial dysfunction, inflammatory reaction, and PCD regulation (Zhang S. et al., 2024). WS and WA entail an intricate interplay of mechanisms against MIRI, including alleviating oxidative stress and suppressing apoptosis (Figure 7).

#### 4.2.1 Alleviating oxidative stress

Findings reported revealed potent anti-oxidative stress effects of WS and WA against MIRI (Gupta et al., 2004; Mohanty et al., 2008; Yan et al., 2018). Relative to the IR control group, oral treatment with WS extract restored myocardial oxidant-antioxidant balance in healthy experimental rats and markedly augmented the levels of GSH, SOD, CAT, glutathione peroxidase (GSHPx) (Gupta et al., 2004; Mohanty et al., 2008). In contrast, vitamin E, as the positive control, failed to induce endogenous antioxidant synthesis (Gupta et al., 2004). Accumulated data also demonstrated that WA could alleviate excessive oxidative stress by elevating the expression of superoxide dismutase 2/3 (SOD2/3), and Peroxiredoxin-1(Prdx-1), whereas supplementation with Akt inhibitor IV markedly reversed the oxidative stress inhibition mediated by WA (Yan et al., 2018).

#### 4.2.2 Suppressing apoptosis

The data showed that WS prior-treatment exerted marked anti-apoptotic effects through modulating the balance between the anti-apoptotic protein Bcl-2 and the pro-apoptotic protein Bax (Mohanty et al., 2008). In addition, the ability of WA to enhance the survival of H9c2 cells and resist apoptosis induced by simulated I/R (SI/R) or hydrogen peroxide (H_2_O_2_) stimulation. Additionally, WA regulated cardiomyocyte caspase-3 activity via an Akt-dependent mechanism, verifying that WA inhibits apoptosis through Akt activation (Yan et al., 2018). Moreover, Guo et al. evaluated cardiac function in wild-type and AMP-activated protein kinase domain-negative (AMPK-DN) transgenic mice following intervention with two distinct WA doses (1 mg/kg and 5 mg/kg), so as to explore the cardioprotective effects of WA in MIRI (Guo et al., 2019). Cardioprotective outcomes were observed in the low-dose WA (1 mg/kg) group rather than the high-dose (5 mg/kg) group. The cardioprotective mechanism of WA involves AMPK-dependent upregulation of the mitochondrial anti-apoptotic pathway (Guo et al., 2019).

### 4.3 Effects and mechanisms of WA against RIRI

RIRI is a pathological process that can lead to various retinal disorders, such as glaucoma, diabetic retinopathy, and retinal vascular occlusion (Minhas et al., 2016). Currently, there has been only one study on the treatment of RIRI with WA. The results showed that WA can increase the production of key antioxidant molecules HO-1 and Prdx-1 during RIRI through activating the Akt signaling pathway (Yan et al., 2022). Moreover, WA activated the Akt signaling pathway and decreased the apoptosis of human retinal microvascular endothelial cells (HRMECs) (Yan et al., 2022). The blockade of Akt completely abolished the effects of WA, suggesting that WA can ameliorate RIRI by reducing endothelial cell apoptosis via the Akt-dependent Prdx-1 and HO-1 antioxidant signaling pathways (Yan et al., 2022).

## 5 Discussion and future prospects

I/R injury is a pervasive and intractable pathological process in clinical practice, occurring extensively in high-metabolic organs. Although reperfusion serves as a key intervention to restore blood flow and rescue tissue function, the resulting secondary injury is often more severe than the initial ischemia. In recent years, with in-depth investigations into the mechanisms underlying I/R injury, the academic community has gradually recognized that I/R injury essentially involves a complex network encompassing oxidative stress, calcium overload, mitochondrial dysfunction, inflammatory responses, and various forms of programmed cell death. Single-target intervention strategies are therefore difficult to achieve the desired therapeutic effects. Consequently, holistic regulatory strategies targeting multiple molecules and pathways have emerged as a new direction for the prevention and treatment of I/R injury.

TCM exhibits unique advantages in the management of complex diseases, attributed to its “multi-component, multi-target, and multi-pathway” characteristics according to the “holistic concept” and “syndrome differentiation and treatment.” WS, a classic herb in Ayurvedic medicine, has attracted considerable attention in recent years, particularly in the field of I/R injury research. WA, one of its active components, has been demonstrated to possess significant potential in mitigating I/R injury. This article systematically reviews the protective effects and mechanisms of WS and WA in I/R injury, including CIRI, MIRI and RIRI though WS and WA started relatively late in the field of I/R injury research.

Firstly, WS and WA have shown good safety profiles in multiple animal models. In acute toxicity studies, the NOAEL for both WS and WA could reach up to 2,000 mg/kg, which is substantially higher than the experimentally effective dose. Subacute and chronic toxicity studies have also failed to reveal any significant toxic effects. Moreover, WS is used in a variety of products, which are distributed and sold in most world markets and usually appear in the form of dietary supplements (Brendler et al., 2025). WA is identified as a potential drug candidate with the potential to be developed into a therapeutic agent based on its physical and chemical properties as well as structural characteristics (Yan et al., 2022). Pharmacokinetically, WS and WA exhibited moderate-to-high oral bioavailability and rapid clearance (Hassannia et al., 2020), suggesting that the favorable pharmacokinetic properties and low toxicity of WS and WA make them promising candidates for further development.

Based on current preclinical evidence, the protective effects of WS and WA against CIRI, MIRI and RIRI are not mediated by a single pathway, but rather through the synergistic actions of multiple mechanisms. In CIRI, WS exerts comprehensive protective effects, including antioxidation, anti-inflammation, anti-apoptosis and anti-mitochondrial dysfunctions, promotion of neural regeneration and inhibition of VSMC migration, respectively,and these protective mechanisms are closely related to the PARP1-AIF, PI3K/Akt and NF-κB pathways. In MIRI, WA upregulates SOD2, SOD3, and Prdx-1 via an Akt-dependent mechanism, thereby suppressing oxidative stress and concurrently reducing caspase-3 activation to exert anti-apoptotic effects. Furthermore, low-dose (1 mg/kg) WA enhances mitochondrial anti-apoptotic capacity through an AMPK-dependent pathway. However, high-dose (5 mg/kg) WA exhibits the opposite effect, demonstrating that WA has a significant hormesis or off-target toxicity. Given that this dose-effect relationship directly affects the clinical application prospects of WA, its mechanism clarification and safety window assessment in subsequent studies require urgent priority attention. In RIRI, WA can reduce endothelial cell apoptosis through the Akt-dependent Prdx-1 and HO-1 antioxidant signaling pathways, thereby improving RIRI. It is worth noting that these pathways do not operate independently but have complex interactions. For example, Akt is regarded as the upstream signaling molecule of HO-1 in most studies. The main mechanism is that Akt activates the nuclear factor erythroid-2 related factor 2 (Nrf2), thereby promoting the gene expression of HO-1 and subsequently inducing antioxidation responses (Fang et al., 2024; Wang et al., 2016). Meanwhile, Akt is not only a core transcription factor in the oxidative stress response but also regulates the expression of anti-apoptosis and anti-inflammation. The PI3K/Akt pathway not only inhibits apoptosis but also negatively NF-κB-mediated pro-inflammatory response (Liu et al., 2020; Deng and Zhou, 2024). Therefore, the synergistic regulation of WS and WA on these pathways is the molecular basis for its anti-I/R injury effect.

Due to the heterogeneity in their physiological functions, cell composition, metabolic characteristics, and microvascular structures, the susceptibility and response degree of various organs to I/R injury vary. Through the summary of the above studies, the common mechanism of WS and WA in protecting against I/R injury was elucidated by inhibiting oxidative stress and anti-apoptosis, which directly target the two core pathological links of ischemia/reperfusion injury. However, it is worth noting that the pathological network of I/R injury also includes calcium overload, inflammatory cascade reactions, and non-apoptotic programmed cell death pathways. Therefore, the interventional effects of WS and WA have not been fully elucidated and warrant systematic investigation, which holds significant scientific value and broad research potential. All in all, these findings suggest that the protective effects of WS and WA in I/R injury are not accidental, but rather determined by their multi-target pharmacological properties. Figure 9 illustrated the mechanism of WA and WS in resisting I/R injury.

**FIGURE 9:**
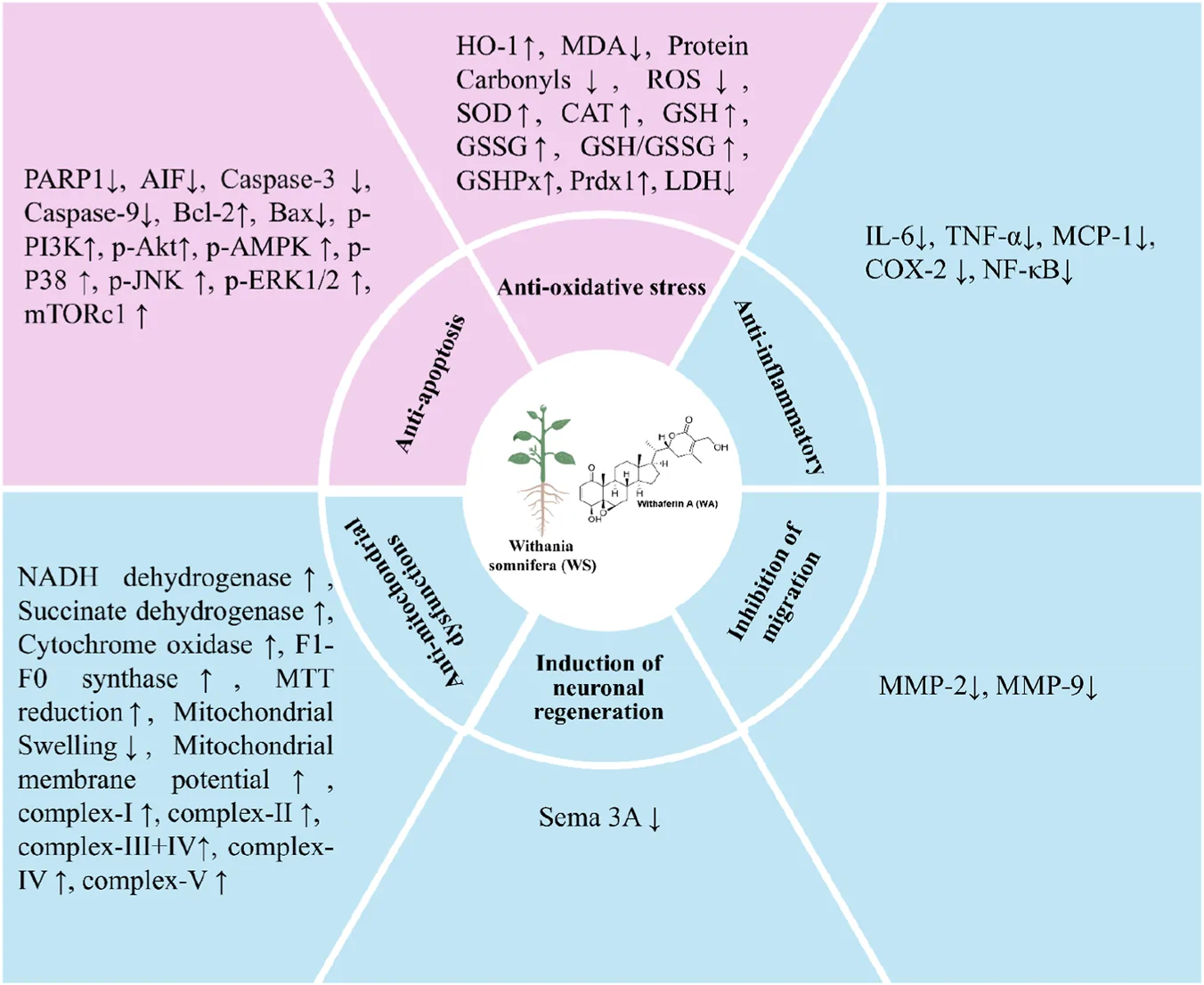
The anti-I/R injury activity of WS and WA. The magenta-colored blocks represent the common protective mechanisms, including anti-apoptosis and antioxidant stress, while the light blue blocks represent the other protective mechanisms specific to WS or WA for anti-CIRI.

Despite the considerable potential of WS and WA in anti-I/R injury research, current studies remain predominantly at the preclinical stage, and multiple challenges must be addressed before their widespread clinical application. Based on the systematic review herein, the following suggestions for future research directions are proposed: (1) Currently, the research involves multiple animal models, cell lines, and I/R injury modeling schemes, which leads to significant heterogeneity in experimental conditions, thereby reducing the comparability and integration value of the results. Moreover, several original studies did not meet higher quality standards, but due to the limited number of existing literature, these studies were not excluded. (2) The existing research designs tend to be simplified, reporting a large number of signaling pathways, and the mechanism studies are relatively simple, lacking in in-depth exploration such as gene knockout/overexpression or tissue-specific intervention, and the regulatory network among signaling pathways is still unclear. (3) Studies only focuses on the biological effects of different doses of WS or WA, and did not use positive controls for comparison, which restricted the comprehensive evaluation of the relative efficacy and safety of WS and WA. (4) The pharmacokinetic data of WS and WA on I/R injury are very limited, especially no studies have investigated the urinary or fecal excretion of WS or WA. (5) Our study mainly focuses on the anti-I/R injury protection effects of WS and WA, however, it does not rule out the possibility that other monomers (such as NMITLI-118 and withanolide A) also have potential protective activities against CIRI (Ahmad et al., 2016; Mukherjee et al., 2020), suggesting that WS may contain other active components with synergistic effects. Nevertheless, the current research is still at an early stage and the literature reports are extremely limited. Therefore, this direction will become an important research topic in the future. Therefore, future research should first focus on establishing a standardized experimental system, unifying core parameters such as animal strains, ischemia-reperfusion time windows, etc., and developing a fully validated highly sensitive UHPLC-MS/MS method to systematically determine the pharmacokinetic and excretion characteristics of free/combined forms of WA and WS in urine and feces. Meanwhile, positive controls should be added to conduct head-to-head comparative studies to scientifically define the relative efficacy and safety boundaries of WS and WA. In terms of mechanism exploration, it is necessary to rely on tintegrate single-cell sequencing and phosphorylation proteomics technologies, reconstruct the temporal cross-regulatory network between signaling pathways, and clarify the key targets and their causal relationships. In addition, it is necessary to break away from the single-component research paradigm and actively expand to other potential active monomers such as NMITLI-118 and withanolide A. It is necessary to conduct in-depth research on the synergistic or additive effects among various components, and to construct multi-component pharmacokinetic/pharmacodynamic (PK/PD) models. The above multi-dimensional and cross-scale integrated research will lay a solid theoretical and experimental foundation for WS and WA as candidate drugs for I/R injury adjunctive therapy, and further actively promote their transition to clinical application.

## 6 Conclusion

In summary, WS and its bioactive constituent WA exert prominent protective effects against organ I/R injury. Such protective effects are mediated through antioxidant, anti-inflammatory, and anti-apoptotic activities, mitochondrial function modulation, and coordinated regulation of multiple signaling cascades. The current research is limited to studies at the cellular and animal levels and lacks sufficient clinical translation evidence. More interdisciplinary and multi-dimensional clinical studies will accelerate the scientific transformation and clinical application of traditional Chinese medicine in modern medical practice in the future.

## Glossary

ADME: Absorption, distribution, metabolism, and excretion
AIF: Apoptosis-inducing factor
Akt: Protein kinase B
AMPK-DN: AMP-activated protein kinase domain-negative
ASC: Apoptosis-associated speck-like protein
BBB: Blood–brain barrier
CAT: Catalase
CIRI: Cerebral ischemia-reperfusion injury
COX-2: Cyclooxygenase-2
CYP450: Cytochrome P450
DAMPs: Damage-associated molecular patterns
ETC: Electron transport chain
FDA: Food and Drug Administration
GPX4: Glutathione peroxidase 4
GSDMD: Gasdermin D
GSH: Glutathione
GSHPx: Glutathione peroxidase
**H**_**2**_ **O**_**2**_: Hydrogen peroxide
HIF-1α: Hypoxia-inducible factor 1α
HMGB1: High-mobility group box protein 1
HO-1: Heme oxygenase-1
HRMECs: Human retinal microvascular endothelial cells
I/R: Ischemia/reperfusion
IL-1β: Interleukin-1β
IL-6: Interleukin-6
**LD**_**50**_: Median lethal dose
LMW-SHs: Low molecular weight thiols
MCAO: Middle cerebral artery occlusion
MCP-1: Monocyte chemoattractant protein-1
MIRI: Myocardial ischemia-reperfusion injury
MMP: Matrix metalloproteinase
mPTP: Mitochondrial permeability transition pore
NAK: Na^+^/K^+^-ATPase
NCX: Na^+^–Ca^2+^ exchanger
NF-κB: Nuclear factor-κB
NHE: Na^+^–H^+^ exchanger
NOAEL: No observed adverse effect level
Nrf2: Nuclear factor erythroid-2 related factor 2
PARP1: Poly (ADP-ribose) polymerase-1
PCD: Programmed cell death
PDGF: Platelet-derived growth factor
PI3K: Phosphoinositide 3-kinase
pMCAO: Permanent distal middle cerebral artery occlusion
Prdx-1: Peroxiredoxin-1
PRRs: Pattern recognition receptors
P-SHs: Protein thiols
RET: Reverse electron transfer
RIRI: Retinal ischemia-reperfusion injury
ROS: Reactive oxygen species
Sema3A: Semaphorin-3A
SI/R: Simulated I/R
SOD: Superoxide dismutase
TCMs: Traditional Chinese medicines
TLRs: Toll-like receptors
TNF-α: Tumor necrosis factor-α
T-SHs: Total thiols
VSMC: Vascular smooth muscle cell
WA: Withaferin A
WS: *Withania somnifera*
WSC: WS root extract
